# Upper Extremity Proprioception in Healthy Aging and Stroke Populations, and the Effects of Therapist- and Robot-Based Rehabilitation Therapies on Proprioceptive Function

**DOI:** 10.3389/fnhum.2015.00120

**Published:** 2015-03-02

**Authors:** Charmayne Mary Lee Hughes, Paolo Tommasino, Aamani Budhota, Domenico Campolo

**Affiliations:** ^1^Robotics Research Centre, School of Mechanical and Aerospace Engineering, Nanyang Technological University, Singapore; ^2^Interdisciplinary Graduate School, Nanyang Technological University, Singapore

**Keywords:** proprioception, stroke, aging, rehabilitation robotics, upper extremity

## Abstract

The world’s population is aging, with the number of people ages 65 or older expected to surpass 1.5 billion people, or 16% of the global total. As people age, there are notable declines in proprioception due to changes in the central and peripheral nervous systems. Moreover, the risk of stroke increases with age, with approximately two-thirds of stroke-related hospitalizations occurring in people over the age of 65. In this literature review, we first summarize behavioral studies investigating proprioceptive deficits in normally aging older adults and stroke patients, and discuss the differences in proprioceptive function between these populations. We then provide a state of the art review the literature regarding therapist- and robot-based rehabilitation of the upper extremity proprioceptive dysfunction in stroke populations and discuss avenues of future research.

## Introduction

Proprioceptive information is important for balance and postural control, the control and regulation of coordinated movements, motor learning, and error correction during movements (Jeannerod, 1988; Schmidt and Lee, 1988) and is generally composed of the modalities joint position sense and the sensation of limb movement (Gandevia et al., 2002). Joint position sense is defined as the ability of an individual to identify the static location of a body part, and is served by muscle spindle afferents and cutaneous afferents (Proske, 2006; Proske and Gandevia, 2009). Kinesthesia, a term introduced by Bastian (1887), refers to the perception of active and passive motion. Passive motion sense is served by slowly adapting mechanoreceptors (mainly secondary spindle endings and tendon organs in muscle), and tendon organs and Ruffini spray endings in other deep tissues, whereas active motion sense stems from the more rapidly adapting proprioceptors; mainly the muscle spindle primary endings, and lamellated corpuscles in other deep tissues (Grigg, 1994; Hogervorst and Brand, 1998).

The importance of proprioception in performing coordinated movements has been demonstrated in studies investigating motor control in individuals with proprioceptive deficits resulting from sensory neuropathy conditions or surgery (Rothwell et al., 1982; Ghez et al., 1995; Gordon et al., 1995; Messier et al., 2003; Sarlegna et al., 2006) and by disrupting proprioception in physically and neurologically healthy participants using tendon vibration (Cody et al., 1990; Cordo et al., 1995). Deficits in upper extremity proprioceptive function have also been reported in normally aging older adults (Adamo et al., 2007; Riberio and Oliveria, 2007) and individuals with stroke (Twitchell, 1951; Carey et al., 1993; Yekutiel, 2000), and have been found to negatively impact the quality of daily life and independence of the affected individual (Carey et al., 1997).

In this review, we first provide an overview of the behavioral research on upper extremity proprioceptive deficits in normally aging older adults, and then present an up-to-date overview of the proprioceptive declines in stroke patients. We conclude this review by reporting the state of the art in conventional and robotic rehabilitation of upper extremity proprioceptive function, and discuss the existing problems in this field and what may be proposed to move this area of science forward.

## Proprioceptive Function in Healthy Older Adults

Proprioceptive declines in older adults are a result of anatomical and physiological changes in both the central and peripheral nervous systems (CNS and PNS, respectively), which negatively influence the ability to execute everyday tasks in the absence of vision. Changes in the peripheral nervous system that account for declines in proprioceptive function include increases in capsular thickness (Swash and Fox, 1972), decreases in muscle spindle sensitivity (Miwa et al., 1995; Burke et al., 1996; Kim et al., 2007) and diameter (Kararizou et al., 2005), and a lower total number of joint mechanoreceptors [especially for Ruffini, Pacinian, and Golgi-tendon type receptors, Morisawa (1998), Aydog et al. (2006)], intrafusal (Swash and Fox, 1972; Liu et al., 2005), and chain fibers (Liu et al., 2005).

Central nervous system changes include decreased gray matter volume in the anterior cingulate cortex, pre- and postcentral gyri, insula, and angula gyri (Good et al., 2001) and reduced activity in proprioceptive regions of the basal ganglia (Goble et al., 2012), both of which may contribute to declines in joint position sense in older adults. For example, Goble et al. (2012) used magnetic resonance imaging (fMRI) and tendon vibration to stimulate muscle spindle afferents in healthy young (mean age = 26.1 years; range = 19.9–32.4 years) and elderly adults (mean age = 68.9 years; range = 62.3–81.3 years), and reported a localized underactivation of the right putamen in elderly individuals. Using diffusion tensor imaging (DTI), older (but not younger) adults with higher mean fractional anisotropy (FA) were found to have increased right putamen neural activity and better joint position sense performance. On the basis of these results, the authors argued that proprioceptive processing in the elderly is influenced by structural differences that limit activation within subcortical regions (i.e., putamen), which, in turn, influence performance in tests of joint position sense.

Age-related declines in cognitive and sensorimotor processing ability (D’Esposito et al., 1995; Seidler et al., 2010) are also thought to contribute to changes in proprioceptive function, especially in more cognitively demanding tasks. This is supported by studies demonstrating age-related changes in elbow joint position sense in tasks with greater memory demands (Adamo et al., 2007, 2009). In these studies, a contralateral remembered matching task, an ipsilateral remembered matching task, as well as a contralateral concurrent matching task was employed. In the former two tasks, the joint is passively moved to a target position and held for a few second before being returned to its starting angle. Participants are then required to match the memorized target joint angle using the ipsilateral (ipsilateral remembered matching task) or contralateral arm (contralateral remembered matching task). In the latter task (contralateral concurrent matching task), the hand is moved to a target position, and held there while the participant attempts to match the target position with the other hand. Overall, significantly poorer performance was observed in the older adults [mean age = 75.0 years in Adamo et al. (2007), 76.4 years in Adamo et al. (2009)] relative to their younger counterparts [mean age = 27.0 years in Adamo et al. (2007), 22.1 years in Adamo et al. (2009)], with the elderly group exhibiting greater matching errors for the task that required both memory-based matching and interhemispherical transfer of proprioceptive information (i.e., contralateral remembered). On the basis of these results, the authors postulated that the decrease in proprioceptive acuity in elderly individuals reflects age-related deterioration in cognitive function, and is exacerbated in tasks with greater memory demands.

Despite the well documented changes in the CNS, PNS, and neuromuscular systems, there is no general consensus regarding *general* proprioceptive abilities in elderly individuals. The majority of available research examining joint position sense has reported that individuals aged between 70 and 80 years perform much worse than individuals in their 20s and 30s, regardless of whether the movement is performed with the elbow (Adamo et al., 2007; Herter et al., 2014), arm (Stelmach and Sirica, 1986), wrist (Adamo et al., 2009), or finger (Ferrell et al., 1992; Kalisch et al., 2012).

In contrast, age-related declines in the perception of active and passive motion (kinesthesia) have been found in some studies (Ferrell et al., 1992; Wright et al., 2011), but not in others (Kokmen et al., 1978; Wang et al., 2012). For example, Kokmen et al. (1978) reported that passive motion perception at the metacarpophalangeal (MCP) sensitivity was impaired in older individuals (age range = 61–84 years) compared to young adults (age range = 19–34 years), but that when the older cohort was separated into semidecades all differences of motion threshold perception lost significance. Similarly, Wang et al. (2012) reported that older participants (mean age = 66.5 years, range = 61–70 years) showed nearly identical performance to the younger cohort (mean age = 25.0 years, range = 22–29 years) when performing whole arm passive or active movements in the absence of visual feedback. Wright et al. (2011), however, found that the ability to perceive passive wrist movement is altered in older individuals, with passive movement detection thresholds were reportedly twice as high in older adults (mean age = 79.5 years) compared to younger individuals (mean age = 24.0 years).

Considering all available data, there is strong evidence that joint position sense acuity decreases with age. In contrast, the presence of age-related declines in kinesthesia depends on a number of factors (e.g., experimental paradigm and task, how proprioception was measured, whether the task required movement at a single joint or multiple joints). For example, whole arm movements require the integration of sensory signals from a greater number of muscles spanning different joints of different segment lengths in order to accurately estimate joint position sense. Thus, movements that require the whole arm [e.g., Wang et al. (2012)] are likely to be more complex than those than involve a single joint [e.g., Wright et al. (2011)]. More focused examination of task differences may be helpful in elucidating changes in kinesthesia across the life span.

Despite the wealth of research demonstrating that upper extremity joint position sense is worse in individuals aged between 70 and 80 years compared to individuals aged between 20 and 30 years, and that a sedentary lifestyle accelerates loss of joint position sense acuity in older individuals (Adamo et al., 2009), we were unable to find any studies that evaluated the effects of upper extremity proprioceptive training on healthy elderly individuals or any commercial robotic proprioceptive assessment and training systems that cater to elderly individuals. This is intriguing the ample evidence demonstrating the importance of proprioceptive feedback on many daily activities, and that lower extremity proprioception interventions (e.g., posture training and Tai Chi) are associated with increased proprioceptive acuity in active older adults (Sinaki and Lynn, 2002; Li et al., 2008). The examination of upper extremity training on proprioceptive declines is an avenue of research certainly worthwhile pursuing as the early diagnosis and effective management of sensorimotor control and dysfunction provides the opportunity for older adults to enjoy their later years as functional, active, independent members of the community. Researchers and clinicians should consider age-related declines in tactile discrimination and haptic sensation when designing training interventions for the elderly. For example, Stevens and Choo (1996) has reported that tactile acuity thresholds (as measured by two-point discrimination test) in the finger are on average about 80% higher in elderly adults (65 years of age and older) compared to younger adults (between the ages of 18 and 28 years), and that the ability to discriminate tactile gaps, orientation of lines, and the length of lines drawn on the skin (i.e., graphesthesia) deteriorates with age, with approximately 1% per annum increase in threshold between the ages of 20 and 80 years (Stevens and Patterson, 1995; Stevens and Cruz, 1996). Moreover, intervention programs should consider the factors that affect proprioception in elderly and stroke populations, keeping in mind that proprioceptive function may not be fully restored. As such, programs should also facilitate the development of compensatory strategies to ensure safety in both familiar (e.g., home and work) and novel (i.e., shopping center, public transport depot) environments.

## Proprioceptive Function in Stroke Patients

Sensory and proprioceptive deficits are particularly common following stroke, and are correlated with length of hospitalization, likelihood of discharge, and increased mortality rates (Zeman and Yiannikas, 1989; Carey et al., 1993; Carey, 1995; Yekutiel, 2000; Sommerfeld and von Arbin, 2004), and have detrimental effects on personal safety and leisure activity levels after hospital discharge (Carey et al., 1997).

Impairments can range from disruption of one type of sensation modalities (e.g., primary tactile senses such as light touch, pressure and localization to more discriminatory senses, sharp/dull discrimination, temperature discrimination, and proprioception) to impairments of multiple or all somatosensory modalities (Jongbloed, 1986; Carey, 1995; Winward et al., 1999). It has been reported that tactile impairment is more frequent than proprioceptive impairment (Winward et al., 2002; Tyson et al., 2007). For example, Tyson et al. (2007) measured tactile and proprioceptive sensation in 93 acute stroke patients (range: 2–4 weeks post-stroke) using the Rivermead assessment of somatosensory performance (RASP), and reported that tactile impairment (66%) was more common than proprioceptive (27%), and impairment of discrimination was more common than detection (65 vs. 45%). Winward et al. (2002) also used the RASP as a measurement tool in their sample of 100 chronic stroke patients (range: 4.7–6.1 weeks post stroke), and reported impaired surface detection, surface localization, and impaired proprioception rates of 65, 31, and 52%.

In contrast to these two studies, there are reports that prevalence rates of upper extremity proprioceptive impairment are similar (Carey and Matyas, 2011) or greater (Connell et al., 2008) to those obtained for tactile impairment. Carey and Matyas (2011) reported impairments in tactile discrimination and wrist position sense (47 and 49%, respectively) in 51 post-acute and chronic stroke patients (mean days post stroke: 49.5 days). Connell et al. (2008) used the Nottingham sensory assessment (NSA) and found proprioceptive impairment to be more frequent than tactile impairment in a number of upper extremities in their sample of 70 patients (median days post stroke: 15 days), and argued that the discrepancy between studies is due to the use of the RASP in prior studies, which measures joint movement and movement direction discrimination, but not joint position sense, and therefore is less likely to detect proprioceptive impairment compared to the NSA.

Focusing on the modalities joint position sense and kinesthesia, neuroimaging studies have reported proprioceptive impairments after stroke with lesions to the thalamus (Sacco et al., 1987; Ja Gutrecht et al., 1992; Kim, 1992; Lee et al., 2012), posterior limb of the internal capsule [i.e., PLIC, e.g., Shintani et al. (2000)], and somatosensory (S1) and posterior parietal cortices (PPC) (Derouesne et al., 1984; Shintani et al., 2000; Kim, 2007). These brain areas are involved in numerous functions related to sensory processing in the healthy brain (Riehle and Vaadia, 2004). Specifically, the ventral posterolateral (VPL) nucleus of the thalamus projects somatosensory information from the extremities to the cortex, the PLIC transmits ascending sensory signals from the thalamus and descending motor commands from the cortex, S1 receives somatosensory information via VPL, and the superior parietal lobule (SPL) of PPC receives heavy input from S1 and projects to all areas of premotor cortex (with the exception of the ventral bank of the caudal cingulate motor area).

Proprioceptive deficits after stroke have been found to correlate with visuospatial neglect (Vallar et al., 1993, 1995; Semrau et al., 2013), with this combination of deficits, resulting in longer recovery times and poorer functional outcome (Smith et al., 1983). The observed proprioceptive deficits in patients with visuospatial neglect lead to the inference that spatial aspects of both vision and proprioception are damaged, and are supported by recent neurophysiological work demonstrating that the PPC is a common site for processing aspects of both sensory modalities (Buneo and Andersen, 2012).

It has also been shown that both the ipsilateral and contralateral limb (with respect to the side of the lesion) is affected after unilateral hemisphere stroke (Connell et al., 2008; Niessen et al., 2008). The pathophysiological mechanisms, which result in deficits of the ipsilateral upper extremity, are largely unknown. One hypothesis is that damage to the ipsilesional uncrossed descending corticospinal pathways influence the ability to perceive and interpret somatosensory information (Ziemann et al., 1999). Alternatively, it has been suggested that ipsilateral proprioceptive deficits after unilateral hemisphere stroke arise from a disturbance of interhemispheric, transcallosal transfer (Shimizu et al., 2002; Stinear et al., 2007), which indicates that activation of the ipsilateral hemisphere during unilateral upper-limb movements might be related to excitatory or inhibitory effects in the contralateral hemisphere. From a clinical perspective, given the observed proprioceptive deficits to both limbs, the ipsilateral limb (with respect to the side of the lesion, i.e., non-paretic) should not be used as a benchmark during the assessment of proprioceptive function. With respect to the assessment of joint position sense, for example, the measured error in the contralateral remembered or contralateral concurrent matching task may arise from the reference arm, the matching arm, or both. As such, it may be more appropriate to use the ipsilateral remembered matching task to evaluate joint position sense in individuals with unilateral hemisphere brain damage.

Recovery of sensory function after stroke occurs within the first 6 months after the stroke incidence (Smith et al., 1983; Winward et al., 2002; Connell et al., 2008). For example, Winward et al. (2002) examined recovery in eight sensory modalities in acute and sub-acute patients (*n* = 9 in each group). Results of that study showed significant improvements in the recovery of proprioception in the first 6 months of recovery, with five out of nine acute patients achieving 100% on the proprioception sub-test by the end of the first month, and six out of nine sub-acute patients achieving 97–100% in proprioception movement by week 33. Similarly, Connell et al. (2008) reported significant recovery in upper-limb proprioceptive function in the first 4 months after stroke in acute stroke patients (*n* = 70, mean days post stroke = 15).

In contrast, the association between proprioceptive impairment and motor function during the recovery process is unclear, with some studies reporting a significant correlation between the two variables (Kuffosky et al., 1982; La Joie et al., 1982; Sheikh et al., 1983; Wade et al., 1983; Pavot et al., 1986; de Weerdt et al., 1987; Mercier and Bourbonnais, 2004), while other studies have not (Twitchell, 1951; Katrak et al., 1998; Rand et al., 1999). For example, Wade et al. (1983) evaluated 25 prognostic factors and reported significant correlations between motor function recovery of the hemiplegic upper limb and initial motor deficit, loss of position sense in the arm, and the Camden Mental Score. Likewise, Connell et al. (2008) found that initial somatosensory impairment and upper-limb tactile sensation predicted proprioceptive recovery 6 months post stroke, and Sheikh et al. (1983) reported that sensory deficit was a significant predictor of disability in 900 stroke patients that were discharged.

Katrak et al. (1998) on the other hand, found that sensory and proprioceptive function (as measured by light touch, sensory inattention, and proprioception in the hemiplegic upper limb) was not correlated with recovery of hand movement or function (*n* = 71, all <28 days post stroke) over a 3-month period. A later study (Rand et al., 1999) compared upper extremity motor and functional recovery in patients with pure motor hemiplegia with that of patients afflicted by combined motor and proprioception deficits (*n* = 20 in each group, mean days post stroke = 17) during the first 6 weeks of rehabilitation. Results showed that motor and functional recovery improved for both groups across the rehabilitation period, with no observable relationship between the loss of proprioception and motor and functional recovery. Moreover, despite some observable improvement the majority of patients with combined sensory and motor deficits still exhibited moderate to severe impaired proprioception, indicating that the recovery of upper extremity motor function is not affected by proprioceptive loss.

The mixed results regarding the relationship between proprioception and the recovery of upper extremity function may be due to differences in proprioception measures, heterogeneity in study populations, and the number of somatosensory modalities and body areas assessed. Further studies are needed to establish whether proprioceptive impairment influences the recovery of upper extremity function after stroke, as this may have important implications for rehabilitation programs.

## Differences in Proprioceptive Function between Healthy Elderly and Stroke Patients

The biological process of aging contributes to the increased risk of stroke, with individuals aged 65 and over accounting for more than two-thirds of hospitalizations (Hall et al., 2012). As such, some of the mechanisms that underlie proprioceptive dysfunction in people with stroke may be attributable to the natural aging process.

As in healthy older adults, acute and chronic stroke patients exhibit impairments in joint position sense (Niessen et al., 2008; Dukelow et al., 2012; Kattenstroth et al., 2013) and kinesthesia (Niessen et al., 2008). For example, Kattenstroth et al. (2013) reported that hand position sense acuity was significantly lower for sub-acute stroke patients (*n* = 10, mean weeks post stroke = 2.3) compared to healthy age-matched controls. Niessen et al. (2008) also found that shoulder joint position sense performance was lower in both the contralateral and the ipsilateral shoulders of sub-acute stroke patients (*n* = 22) when compared with healthy control subjects, although this effect failed to reach significance (*p*’s ranged from 0.063 to 0.299). This latter study also revealed a significant decrease in contralateral and ipsilateral shoulder kinesthesia (as measured by TDPM) for patients compared with the control group. The authors argued that the kinesthetic deficits exhibited by stroke patients arose from gamma motoneuron control dysfunction due to hemiparesis as a result of a stroke. This dysfunction affects the sensitivity of the muscle spindles, which leads to delays in movement detection when the muscle is stretched passively, hence larger TDPM scores. On the other hand, the large degree of humeral rotation in the joint position sense test (10° of internal or external rotation, relative to the chosen start position) passively stretched and sensitized the muscle spindles prior to reaching the reference position. Sensitizing the muscle spindles in this way afforded the accurate detection of the reference position in both groups.

To date, relatively little is known about the specific association between the location of brain lesions and proprioceptive dysfunction. This is unfortunate given that elucidating this relationship could enhance our understanding of the neural circuitry involved in proprioception and could lead to advances in diagnosis, preventive interventions, and treatment. From the limited number of studies conducted so far it appears that there is no specific association between lesion location and either joint position sense (Niessen et al., 2008; Dukelow et al., 2012; Kattenstroth et al., 2013) or kinesthesia (Niessen et al., 2008). One study (Dukelow et al., 2012) compared upper extremity joint position sense in 100 inpatient stroke rehabilitation subjects (mean age = 63.0, range = 21–90) with that of 231 non-disabled controls (mean age = 48.0, range = 20–88) and reported that a large number of individuals with ischemic stroke of the left (*n* = 17/31) and right (*n* = 24/29) middle cerebral artery (MCA) displayed proprioceptive deficits. However, proprioceptive deficits in participants with MCA lesions did not differ statistically from patients with lesions in other locations (i.e., left pontine artery, left basilar artery, left anterior cerebral artery). That said these results should be interpreted with caution due to lower power (small sample size, heterogeneity of patient groups, the high variability in patient performance), which may influence the ability to detect clinically meaningful effects. Given the importance that elucidating the lesion–symptom relationship could have for rehabilitation outcomes it is recommended that future studies employ sophisticated neuroimaging analysis techniques (Voxel-based morphometry, Voxel-based lesion–symptom mapping) to identify lesion characteristics associated with proprioceptive dysfunction in stroke patients.

## Rehabilitation of Proprioceptive Function in Stoke Populations

### Therapist-based interventions

Sensory interventions to improve function or remediate sensory and proprioceptive impairments of the upper limb following stroke can occur via either passive or activity sensory training. Passive sensory training involves the application of electrical stimulation to produce activation of cutaneous nerves in the absence of muscle contraction, whereas active sensory training involves a series of exercises designed specifically to train sensory function.

There is preliminary evidence that passive sensory training (i.e., via the application of electrical stimulation to produce activation of cutaneous nerves in the absence of muscle contraction) leads to significant improves in tactile sensation (Cambier et al., 2003), kinesthetic sensation (Cambier et al., 2003), light touch detection (Acerra et al., 2005), pressure and temperature pain sensation (Acerra et al., 2005), mechanical sensation (Chen et al., 2005), and arm function (Cambier et al., 2003; Chen et al., 2005) in individual with stroke. For example, Cambier et al. (2003) examined the effects of intermittent pneumatic compression of the hemiplegic upper limb (*n* = 12, mean time since stroke = 83 days) compared to sham therapy (*n* = 11, mean time since stroke = 114 days). Results indicated that somatosensory function improved for both groups over the course of treatment, but the improvements were greater for the group that received standard physiotherapy combined with intermittent pneumatic compression treatment (experimental group) compared to the control group that received sham treatment (81.1 vs. 30.9% improvement). That study also demonstrated between-group differences in tactile (37.10) and kinesthetic sensation (26.20), but not for two-point discrimination (0.31) and stereognosis (5.60), in favor of the experimental group. In sum, intervention protocols utilizing passive sensory training have the potential to improve motor and sensory impairment of the upper limb after stroke. However, this corpus work is still in its infancy and further high quality studies are needed in order to properly evaluate their effectiveness in post-stroke populations.

Researchers have also examined the effects of active sensory training programs on stroke recovery (see Table 1). The first known study to examine the effects of re-education and training on upper extremity proprioceptive function after stroke was conducted by Carey et al. (1993) (Exp 2). In that study four patients (time since stroke = 5–26.5 weeks) completed a 30 session training program, which consisted of graded texture (tactile discrimination test, TDT) and limb position discrimination training (proprioceptive discrimination test, PDT). Results indicated that patients showed significant improvements of trained abilities, with discrimination capabilities of the affected hand reaching levels comparable to those of the unaffected hand, which were maintained at 2- and 5-month follow-up. Unfortunately, the clinical relevance of this study was undermined by the fact that the stimuli used in the training program and assessment were identical, and outcome measures were limited to two trained submodalities.

**Table 1:** **Characteristics of studies that have examined the effects of upper extremity proprioceptive interventions on stroke function**.

	Carey et al. (1993) (Exp 2)	Yekutiel and Guttman (1993)	Smania et al. (2003)	Carey et al. (2011)	Byl et al. (2003)
**Sample size**	4	Experimental group 20 Control group 19	4	Experimental group 24 Control group 25	Group A^a^ 8 Group B^b^ 10
**Age** Mean (SD)	46.5 (19.2)	Experimental group 64.0 (b/w 44–81) Control group 67.0 years (n/a)	51.7 (13.4)	Experimental group 61.08 (14.4) Control group 60.96 (11.2)	Group A 69.0 (5.1) Group B 58.5 (9.6)
**Gender** (M:F)	3:1	Experimental group 13:7 Control group 8:11	2:2	Experimental group 17:8 Control group 20:5	Group A 5:3 Group B 7:3
**Time since stroke** Mean (SD)	12.5 (9.7) weeks	Experimental group 6.2 years Control group 6.2 years	10.0 (6.9) months	Experimental group 32.6^c^ (n/a) weeks Control group 51.9^c^ (n/a) weeks	Overall 30 months Group A (n/a) Group B (n/a)
**Effector**	Wrist	Elbow	Wrist, metacarpophalangeal joint	Wrist	Upper limb
**Proprioceptive** **Intervention**	TDT, PDT	Letter tactile recognition (identification of the number of touches or shapes drawn on the arm and hand), shape, weight, and texture discrimination, kinesthesia (indicate limb position following passive movement), passive drawing	TDT, PDT, Dannenbaum and Dykes pressure sensation test (modified), weight discrimination test, letter tactile recognition, paper manipulation, motor sequence performance, reaching and grasping, thumb-index grip force control, functional ADL tasks	Fabric matching test, TDT, wrist and finger PDT, Roylan hot and cold temperature discrimination kit for finger and forearm, functional tactile object recognition test	Graphesthesia (replicate figures drawn on fingers), touch localization (touch subject and have them identify the area touched), stereognosis (interpret information about an object through exploration with glabrous aspects of the digit), kinesthesia (indicate limb position following passive movement)
**Duration**	30 Sessions	45 min/sessions three times/week for 6 weeks	30 Sessions, each of 50 min duration + 1 h/day home exercises	10 Sessions, each of approximately 60-min duration, conducted three times a week	1.5 h/week For 8 weeks + home gloving of the unaffected hand
**Control condition**	n/a	n/a	n/a	Repeated non-specific exposure to sensory stimuli (that varied in texture, shape, size, weight, hardness, and temperature) via grasping of common objects, and passive movements of the upper limb	Fine motor task practice (e.g., writing, drawing, object manipulation, placing the hand on moving surface to develop graded control), perform general aerobic, strengthening, and flexibility exercises
**Measures**	Same as intervention, but with differences in stimuli and/or conditions	Touch localization (touch subject and have them identify the area touched), PDT, finger, palm and forearm 2-PD, tactile object recognition (identify a fixed series of 20 ADL objects)	Same as intervention, but with differences in stimuli and/or conditions	SSD index derived from scores of texture discrimination, limb position sense, and tactile object recognition	Kinesthesia sub-test of SIPT, graphesthesia sub-test of SIPT, BCB test for stereognosis, digital reaction time, PPB, manual muscle test, ROM, WMFT, CFE
**Findings**	Improvements of affected hand performance that reached levels of the unaffected hand. Effects were maintained at 2- and 5-month follow-up	Large gains in all sensory tests for the experimental group. Improvements in kinesthesia did not correlate with any other tests, and were negatively correlated with sensory score at outset	Improvements in JPS were reported in three patients. Effects were maintained at 6-month follow-up	Greater improvement in sensory capacity following sensory discrimination training. Effects were maintained at 6-week and 6-month follow-up	Improvements in performance were measured in proprioception after training for both groups, with >21% gains in proprioceptive performance in 83% of patients

*^a^Sensory training 4 weeks, motor training 4 weeks (Group A)*.

*^b^Motor training 4 weeks, sensory training 4 weeks (group B)*.

*^c^Median values provided*.

*2-PD, Two-point discrimination; BCB, Byl-Cheney-Boczai test; CFE, California functional evaluation; JPS, joint position sense task; PDT, proprioceptive discrimination test; PPB, Purdue Peg Board task; ROM, range of motion; SIPT, sensory integration praxis test; SSD, standardized somatosensory deficit; TDT, tactile discrimination test; WMFT, wolf motor function test*.

More recent studies (Yekutiel and Guttman, 1993; Smania et al., 2003) used experimental protocols in which the training activities differed from those used to assess somatosensory function, and also assessed a number of submodalities. For example, in Yekutiel and Guttman (1993) 20 patients (all >2 years post stroke) with chronic hemiplegia and somatic deficits received retaining of sensory function three times/week for 6 weeks (with each session lasting approximately 45 min). The training program consisted of the modalities letter tactile recognition, shape, weight, and texture discrimination, kinesthesia, and passive drawing. Overall, the treated group showed large and significant gains in all sensory tests, compared to a control group of similar age. However, gains in elbow position sense were not correlated with those of any of the other three tests, and were also negatively correlated with sensory score at outset.

Smania et al. (2003) evaluated the effects of a training program that targeted somatic sensation as well as sensory-related deficits in motor control in four unilateral chronic stroke patients (time since stroke = 5–20 months). The training program consisted of thirty treatment sessions each lasting 50 min. At the beginning of each training session patients performed a series of 25 manual exercises, after which the therapist selected the more challenging manual exercises that were then performed with the impaired hand. Despite differences in the degree of improvement between patients, significant improvements in a number of sensory and motor tasks were reported, which were maintained at 6-month follow-up. Results of this study indicate that proprioceptive function can be improved through training programs targeting somatosensory and related deficits of motor control, and that training generalizes to activities not targeted by the training program.

Carey et al. (2011) recently compared the effectiveness of two training programs in fifty chronic stroke patients (all >6 weeks post stroke) with impaired texture discrimination, limb position sense, and/or tactile object recognition. In the sensory discrimination program (SD group), participants received generalized texture discrimination (TDT and fabric matching test), limb position sense (wrist and finger PDT), tactile object recognition (functional tactile object recognition test), and temperature discrimination training (Roylan hot and cold temperature discrimination kit). In contrast, the non-specific training program (NS group) consisted of non-specific repeated exposure to stimuli varying in texture, shape, size, weight, hardness, and temperature, via grasping of common objects, and passive movements of the upper limb. The training program consisted of 10 intervention sessions, each of approximately 60-min duration, conducted three times a week. Results indicated that there was very little change in functional somatosensory discrimination capacity scores (a composite score comprised of the modalities: texture discrimination, limb position sense, and tactile object recognition) for the majority of patients in the NS group, and those with moderate or severe impairment showed little or no improvement. In comparison, almost all (*n* = 22/25) patients in the SD group showed a positive change in functional somatosensory discrimination capacity due to sensory retraining, and there was no observable difference in the magnitude of change between different levels of impairment severity. Proprioceptive improvement was maintained and slightly increased at 6-week and 6-month follow-up. The results of this randomized controlled trial [National Health and Medical Research Council (NHMRC) level II], therefore, indicate that chronic stroke patients with proprioceptive deficits can benefit from sensory discrimination training.

The first study to evaluate the effects of a sensory training on kinesthesia in stroke patients was conducted by Byl et al. (2003). In that study, chronic stroke patients (*n* = 18, time since stroke = 6 months to 7 years post stroke) were randomly assigned to Group A (*n* = 8, sensory training 4 weeks, motor training 4 weeks) or Group B (*n* = 10, motor training 4 weeks, sensory training 4 weeks). The motor training program consisted of practicing repetitive specific fine motor tasks, while the sensory retraining program consisted of graded and repetitive sensory discrimination activities [e.g., replicating drawings of letters, numbers, or figures drawn on the skin (graphesthesia), touching subject on the digit and hand and have subject put finger on spot where touched (touch localization), grasping and interpreting information about the objects properties (stereognosis), indicating limb position following passive movement (kinesthesia)]. Significant improvements in functional independence and performance parameters of the affected upper limb were reported, with improvement >20% for motor (motor reaction time, Purdue Pegboard Task), sensory discrimination (kinesthesia, graphesthesia, stereognosis), and functional independence (California functional evaluation) scores (27, 21, and 41%, respectively) performance in 83% of patients. The results of this study demonstrate that 12 h of supervised learning based sensory motor training programs are sufficient to achieve significant improvements in function in chronic stroke patients that are maintained at 6-month follow-up.

In sum, the relatively few studies with treatment interventions that specifically target somatosensory recovery after stroke have reported positive improvements in upper-limb proprioceptive function in acute (Carey et al., 1993) and chronic stroke patients (Yekutiel and Guttman, 1993; Byl et al., 2003; Smania et al., 2003; Carey et al., 2011). However, a number of these studies suffer from small sample size issues (*n* < 20 patients; Carey et al., 1993; Smania et al., 2003), which decreases statistical power, and thus negatively affect the ability of detecting a true effect, and only one study was classified as randomized controlled trial (Carey et al., 2011). As such, more high quality (NHMRC level II) studies with homogenous and large sample sizes are needed in order to determine (via meta-analysis and effect size calculations) the overall effectiveness of proprioceptive retraining programs on stroke.

### Robot-based interventions

Current conventional stroke rehabilitation therapies are a labor intensive process, which involves daily one-on-one interactions with therapists that can last for several weeks. The significant burden placed on therapists and healthcare systems has motivated a number of researchers to develop robotic devices targeting post-stroke upper extremity motor rehabilitation [cf. Maciejasz et al. (2014) for an overview for currently developed robotic devices for upper-limb motor rehabilitation]. The advantages of using robots is that they can deliver high-dosage and high-intensity training, interact with human motion, physically assist movement, accurately measure performance, and can provide continual assessment of changes in motor function through performance measures.

The prevalence of proprioceptive impairment in stroke populations has led a number of scientists to develop robotic systems for the quantification and rehabilitation of proprioception function in stroke populations (Casadio et al., 2009; Cordo et al., 2009; Sangueniti et al., 2009; Vergaro et al., 2010; Cho et al., 2014). For example, Cordo et al. (2009) utilized a device fitted with tendon vibrators at the flexor and extensor tendons of the wrist and finger joints to examine the efficacy of the *assisted movement with enhanced sensation* (AMES) approach for proprioceptive rehabilitation. In that study, 18 chronic stroke patients (all >1 year post stroke) performed 30-min daily therapy sessions over a 6-month treatment period. During each session, the robotic device cycled the wrist and fingers in flexion and extension while the patient assisted the motion imposed by device by exerting a flexion force on the device during imposed flexion, and an extension force during imposed extension. At each reversal of movement direction, tendon vibration switched between the flexor and extensor tendons, always applying vibration to the lengthening tendon (i.e., to the muscle antagonistic to the assisted joint motion). Every second day, a joint position test was conducted in which the patient was instructed to follow a graphically presented target while staying inside the target zone. Results showed improvements in the joint positioning task across the 6-month period for all patients (average improvement of 77%), with a recovery trajectory for the majority of patients (*n* = 15/18) following a negative exponential trajectory that reached 90% of asymptote at day 111 (approximately 3.6 months). The results of this study, thus, indicate that stimulating proprioceptive afferents in the lengthening muscles during voluntary contraction improves joint position performance after a 6-month training period.

Serious games are a fundamental component of many robotic rehabilitation devices, which while useful for patient motivation and retention, also allow patients to compensate for proprioceptive deficits by relying on vision. To counteract such compensatory strategies the Robotics, Brain and Cognitive Sciences Laboratory at the Italian Institute of Technology (Casadio et al., 2009; Sangueniti et al., 2009; Vergaro et al., 2010) have developed a training framework that manipulates the amount of visual information available during task performance in order to force the patient to rely on proprioceptive feedback. For example, in Vergaro et al. (2010) ten chronic stroke patients (all <1 year post stroke) were asked to grasp the handle of the Braccio di Ferro robot and track a moving target that drew a figure-of-eight shaped trajectory on the computer screen. In the visuo-haptic (VH) condition, the position of the target was presented to the subjects visually by means of a circle on the computer screen, and haptically via an attractive force field to the target. During the pure haptic (PH) condition, the patient was blindfolded and had to rely on the robot-generated force field her to detect the direction the target was moving. In general, there was a significant decrease in the level of assistive force required, a decrease in tracking error, and an increase in movement smoothness across sessions. Based on these results, Vergaro et al. (2010) argued that training lead to a recalibration of sensory channels, and that patients were capable of performing continuous tracking tasks using only proprioceptive cues.

A recent study utilized virtual reality (VR) technology to assess the effect of proprioceptive feedback in upper extremity rehabilitation in stroke patients (Cho et al., 2014). In that study, 10 patients (all >10 weeks post stroke) interacted with a living room VR environment that featured a semi-transparent cylinder that represented the position of the hand, and an opaque cylinder that represented the target position. In the visual feedback virtual environment (VTFE) condition, patients moved the semi-transparent cylinder (current hand position) to the position of the opaque cylinder (target position), and pressed a mouse button with the unaffected hand once they believed the affected hand reached the target position. In the proprioception feedback virtual environment (PFVE) condition, both cylinders were visible at the start of the trial. However, as soon as the patient initiated the reaching movement the semi-transparent cylinder (reflecting the current hand position) disappeared, forcing the patient to rely on proprioceptive feedback to estimate the current and final position. As with the VFVE condition, patients pressed the mouse button once they believed the semi-transparent cylinder corresponded to the position of the opaque cylinder. Results of this study showed a significant improvement in performance after PFVE training, compared to VFVE training, which suggests that improvements in proprioceptive function post-stroke are greater when rehabilitation training systems force patients to rely on proprioceptive feedback.

## Future Work

This article reviewed the literature concerning upper extremity proprioceptive deficits in normally aging older adults and individuals with stroke. Despite the advances in this field, there are still important areas where greater progress is needed. In the first instance, a clear understanding of the effects of increasing age proprioception is important clinically for identifying the impact of stroke on proprioceptive function. As such, it is important that future studies include age-matched controls of appropriate sample size. In the long term, this data can be used to develop a database of normative proprioceptive function that enables the comparison of measurement values during initial assessment and across the training period.

In addition, more accurate clinical assessment of proprioception is vital. Currently, clinical evaluation of proprioceptive function is typically undertaken in a subjective, non-standardized and unreliable manner, and suffers from very poor inter-rater reliability and sensitivity and poor or absent normal value criteria (Lincoln et al., 1991). In contrast, rehabilitation robots capable of proprioceptive assessment have better diagnostic and prognostic precision and are more likely to discriminate subtle differences of deficit and changes over time compared to current clinical measures (Dukelow et al., 2010, 2012). Moreover, robotic tools affords the opportunity to compare the measurement values obtained immediately after assessment, and allow clinicians to decide the most appropriate treatment options, which should lead to improvements in rehabilitation treatment and lower long term healthcare costs.

With respect to stroke populations, there is preliminary evidence to suggest that conventional and robot-assisted sensory re-education training lead to positive improvement in upper-limb proprioceptive function in acute (Carey et al., 1993) and chronic stroke patients (Yekutiel and Guttman, 1993; Byl et al., 2003; Smania et al., 2003; Casadio et al., 2009; Cordo et al., 2009; Carey et al., 2011; Cho et al., 2014). Despite these results, the number of studies examining proprioceptive dysfunction is small, and future work with large sample sizes is needed to corroborate the observed positive effects. Therapist-based training programs directed at age-related and stroke induced functional losses are limited by manpower and healthcare and insurance policies issues, and as such, rehabilitation robots can be used to alleviate the burden on therapists and healthcare systems. Unfortunately, the examination of proprioceptive recovery after a stroke incidence is still in its infancy, and as such the comparison of proprioceptive recovery rates between therapist-based and robot-based training, and the treatment programs that yield the most effective outcomes, remains an open question.

## Conclusion

This review summarized the most significant research conducted on upper extremity proprioceptive function in healthy older adults and those affected by stroke, with results indicating that proprioceptive function declines as a result of normal aging, and affects between 11 and 75% of stroke patients. The number of stroke patients aged 65 and over accounts for more than two-thirds of hospitalizations, and as such early diagnosis and effective management of sensorimotor control and dysfunction may reduce the risk of stroke. The observed deficits in proprioceptive function in stroke populations have lead a number of researchers to develop therapist-based and robotic proprioceptive training programs. Although this area of research is still in its infancy, these developments should lead to improvements in the evaluation, rehabilitation, and treatment of proprioceptive function.

## Conflict of Interest Statement

The authors declare that the research was conducted in the absence of any commercial or financial relationships that could be construed as a potential conflict of interest.
